# A novel phage encoding host defense modulators demonstrates in-vivo therapeutic efficacy against carbapenem-resistant *E. coli* ST1432

**DOI:** 10.1007/s10096-026-05422-7

**Published:** 2026-02-23

**Authors:** Aylin Uskudar Guclu, Nisanur Ayas, Farzaneh Bonyadi, Sezin Unlu Celebi

**Affiliations:** 1 Faculty of Medicine, Department of Medical Microbiology, Baskent University, Ankara, Türkiye; 2 Faculty of Medicine, Department of Histology & Embryology, Baskent University, Ankara, Türkiye

**Keywords:** Carbapenem-resistant *E. coli*, Bacteriophage, Phage therapy, *Galleria mellonella*, One health

## Abstract

**Aim:**

This study aimed to isolate, identify, characterize, and evaluate in vivo therapeutic efficacy of a lytic bacteriophage against carbapenem-resistant *E. coli* (CREC).

**Method:**

A lytic bacteriophage was isolated from wastewater. The biological properties including morphology, burst size, latent period, optimal MOI, stability of pH and temperature, and host specificity were determined. Its genomic feature was analyzed by whole genome sequencing. The in vivo therapeutic efficacy was evaluated by *Galleria mellonella* infection model against clinical isolate of CREC ST1432 as an emerging One Health MDR threat.

**Result:**

The one-step-growth curve revealed a latent period of 20 min and a burst size of 116 PFU/cell. The phage was stable between 4–60 °C and pH 4–10. The dsDNA genome of Baskent_Phicoli_1 is composed of 39,500 bp with 50.06% GC content. Comprehensive genome analysis confirmed the absence of antimicrobial resistance, virulence, and lysogeny encoding genes. In the untreated infection group (IC), which received a lethal dose of CREC, 73.3% of the larvae died, whereas the survival rate was 100% in both the phage treatment (PT) and toxicity control groups. Extensive melanized nodules were observed in IC, while larvae in PT group exhibited reduced tissue damage in the fat body and muscle layers compared to IC.

**Conclusion:**

This study demonstrated the potential of Baskent_Phicoli_1 as a promising candidate for phage therapy against CREC. Significantly, the phage genome encodes genes for an RNA polymerase inhibitor and an OCR-like anti-restriction protein. The presence of host defense modulators emphasizes the phage's ability to effectively break down bacterial defenses, highlighting its promise for biotechnological applications in combating bacterial infections.

**Supplementary Information:**

The online version contains supplementary material available at 10.1007/s10096-026-05422-7.

## Introduction

The global rise of multidrug-resistant (MDR) bacteria poses a growing public health challenge. Carbapenem-resistant *Enterobacterales* (CRE) are listed by the World Health Organization (WHO) as critical priority pathogens (last access: september 2025) [1]. A key member of this group, carbapenem-resistant *E. coli* (CREC) has increased and is now recognized as a serious clinical threat, associated with infections such as sepsis and urinary tract infections [2, 3]. *E. coli* poses a threat not only in clinical settings but also in environmental, agricultural, and animal reservoirs, where it can persist, acquire resistance genes, and serve as a reservoir for transmission across ecosystem within the One Health context [4]. The dissemination of resistance is driven by the acquisition of carbapenemase encoding genes such as *bla*_OXA-48_, which hydrolyze carbapenems and render them ineffective [5]. With resistance extending even to last-resort antibiotics, CREC infections significantly limit treatment options and are linked to increased mortality, prolonged hospital stays, and higher healthcare costs [2]. These trends highlight the urgent need for novel therapeutic approaches.

Bacteriophages (phages), viruses that infect and lyse bacteria, are reemerging as promising alternatives for antimicrobial therapy [6]. They offer several advantages; high host specificity, self-replication at the site of infection, and a mechanism of action distinct from conventional antibiotics, allowing them to bypass many established antimicrobial resistance mechanisms [7]. On the other hand, to determine their suitability for therapeutic use, phages must be thoroughly characterized in terms of lytic activity, host range, and genomic features [7]. In vivo evaluation of phage efficacy is a key step in translating these agents into clinical practice.

*Galleria mellonella* (wax moth) larvae have gained acceptance as a cost-effective and ethically favorable alternative to mammalian systems for evaluating microbial pathogenesis and antimicrobial activity [8]. These larvae possess a well-developed innate immune system and are simple to maintain, providing a practical experimental model for initial assessment of therapeutic potential in a complex biological environment [9].

The primary objective of this study was to isolate and extensively characterize bacteriophages targeting the clinical isolates of carbapenem-resistant *Escherichia coli* (CREC). It was also aimed to evaluate the phage’s therapeutic efficacy in vivo using *G. mellonella* infection model, to generate foundational insights into its potential as an alternative treatment for CREC infections.

## Material and methods

### Bacterial strains and culture conditions

Clinical isolates of *Escherichia coli* that had been previously determined as CREC carrying *bla*_OXA-48_ encoding genes in previous study were included in the study to be used for phage isolation as a host [10]. The strains were stored in the culture collection of Baskent University Medical Microbiology Laboratory and later cultured on Luria–Bertani (LB) agar (CondaLab, Spain), and incubated at 37 °C for 18–24 h prior to use.

### Bacteriophage isolation, purification, and biological characterization

Water samples were periodically collected from hospital and municipal wastewater. The isolation was performed as described [11] with previous slight modifications [12]. Briefly, water samples were centrifuged at 5000 g for 15 min and 20 ml of the supernatant was transferred to a clean tube containing LB inoculated with the target host bacteria and incubated at 37 °C for 24 h. After incubation, the cultures were centrifuged, and the supernatants were filtered through a 0.22 μm membrane. Then, 10 μL of the phage-containing filtrate was spotted onto the freshly prepared bacterial lawn of the *E. coli* isolate. After 24 h of incubation, the presence of phage cleared the bacterial zone, and the top of the agar layer was picked and soaked in LB. For the purification, a single-plaque isolation procedure was applied, and phage titers were determined by Double-Layer (DL) agar method. The host range of isolated phage was determined by spot test using clinical CREC isolates, and various standard strains including *P. aeruginosa* ATCC 15692, *Klebsiella pneumoniae* ATCC 700603, *Proteus vulgaris* ATCC 13315, *Enterococcus faecalis* ATCC 29212, *Acinetobacter baumannii* ATCC17606, and *Staphylococcus aureus* ATCC 29213.

One-step growth curve analysis was conducted by following the previous modification [13]. A single bacterial colony was inoculated into 10 mL of fresh TSB and incubated overnight at 37 °C, then 500 μL of the overnight culture was transferred into 50 mL of fresh TSB and grown at 37 °C until the logarithmic phase. The 5 mL aliquot of the culture was then centrifuged at 10,000 g for 20 min at 4 °C. The pellet (~10^8^ CFU/mL) was suspended in 500 μL of fresh TSB, mixed with 500 μL of phage suspension (10^7^ PFU/mL), and incubated for 5 min to allow phage adsorption. The mixture was centrifuged again at 10,000 g for 10 min at 4 °C, followed by washing with 10 mL sterile TSB and a second centrifugation step under the same conditions to remove un-adsorbed phages. The final pellet was suspended in 30 mL sterile TSB and incubated at 37 °C. Samples of 100 μL were collected every 10 min, and phage titers were determined by the double-layer agar (DLA) method. Burst size was calculated by dividing the average PFU/mL during the plateau phase by the average PFU/mL of the latent period. The latent period was defined as the time interval between phage adsorption (excluding the 5 min pre-incubation) and the initial burst [14]. All assays were performed in triplicate. Data were plotted using mean values, and standard deviations (SDs) were shown as error bars.

The optimal multiplicity of infection (MOI) was determined by following previous modification [15] by preparing 1 × 10^8^ CFU/mL bacteria and mixing with different titers of bacteriophages to obtain 100, 10, 1, 0.1, 0.01 and 0.001 PFU/CFU. After the incubation at 37 °C for 3.5 h, the samples were centrifuged for 5 min at 10000* g*, and titers were determined by the DL agar method. The MOI that gives the highest titer was determined as the optimal MOI. Temperature (at 4 °C, 10 °C, 20 °C, 30 °C, 40 °C, 50 °C, 60 °C, 70 °C, 80 °C) and pH (ranging from 2 to 12) stability were determined as described before [16] with previous modifications [15]. After the incubation, the number of viable bacteriophages was enumerated by the DL agar method [16].

The morphotype of the isolated phage was determined by transmission electron microscopy (TEM) following a previously described protocol [13] Briefly, high-titer purified phage suspensions (> 10⁸ PFU/mL) were applied (10 µL) onto carbon-coated copper grids (Agar Scientific Ltd., UK) and allowed to adsorb for 5 min. Excess liquid was gently removed using filter paper, and the grids were negatively stained with 2% (w/v) uranyl acetate for 1 min. After air-drying at room temperature, the grids were examined using a TEM (FEI Tecnai, USA) operated at an accelerating voltage of 120 kV. Digital micrographs were captured at appropriate magnifications for morphological characterization.

### Whole genome sequencing and annotation

Nucleic acid extraction was performed using a phenol–chloroform-isoamyl alcohol (25:24:1) from purified phage suspension (10^9^ PFU/ml) according to the previous protocol by [17], and DNA was quantified by a Qubit 4 Fluorometer. Library preparation for Whole Genome Sequencing (WGS) was carried out following Illumina's standard protocol using the NexteraXT DNA Library Preparation Kit (Illumina, USA). Quality and size distribution of the final libraries were assessed using an Agilent 2100 Bioanalyzer (Agilent Technologies, USA). WGS was performed using Illumina NovaSeq 6000 (Illumina, USA) platform. Raw reads were paired and trimmed and de novo assembled using Shovill v. 1.0. FastQC v0.11.9 and Trimmomatic v. 0.39 were applied for determining the quality of the raw data and trimming, respectively. RASTtk v1.073 with default parameters was used for the annotation of open reading frames (ORFs) [18]. Genome sequences of phages were compared at the nucleotide level by BLASTn and demonstrated by Kablammo software [19]. To predict the functions of hypothetical proteins, BLASTp analysis was done against the Non-Redundant Protein Database of NCBI and default parameters [20]. The presence of tRNA-encoding genes was searched by using tRNAscan-SE 2.0 [21]. Presence of the 16S rRNA gene was investigated by using BLASTn against the 16S ribosomal RNA sequences (bacteria and archaea) database. The genome map of the phage was created by cgview. To predict the lifestyle of the phage, BACPHLIP tool was used [22]. The presence of antibiotic resistance, pathogenicity, and virulence genes on the phage genome was investigated by using ResFinder 4.1 [23], virulence factor database (VFDB) [24] and PathogenFinder 1.1 respectively [25]. Whole-genome-based phylogenetic trees was constructed by the VICTOR web service (https://victor.dsmz.de), a method for the genome-based phylogeny and classification of prokaryotic viruses [26]. All pairwise comparisons of the nucleotide sequences were conducted using the Genome-BLAST Distance Phylogeny (GBDP) method [27] under settings recommended for prokaryotic viruses [26]. The intergenomic similarities of the phage was calculated by VIRIDIC [28].

### In vivo experiments

#### Galleria mellonella maintenance and infection model

The therapeutic effect of the isolated lytic phage against CREC isolate was assessed on *G. mellonella* larvae as an in vivo infection model. *G. mellonella* larvae were obtained from local bee farm. To obtain a new generation, the adult moths were maintained in a controlled environment, where they were allowed to mate and oviposit on sterile filter paper inside ventilated containers. Eggs were collected post-oviposition, and the larvae were reared under controlled conditions until they reached the 10th–12th instar stage. Newly hatched larvae were transferred to a sterile container and provided with freshly prepared diet containing; 125 g corn flour, 125 g wheat bran, 62 g milk powder, 70 g molasses, 62 g glycerin, and 30 g yeast [29]. The larvae were maintained at a temperature of 25 °C/30 °C and a relative humidity of 60–70%. To ensure uniformity in physiological state, only freshly emerged larvae from the same cohort were used. All larvae were kept without food in 90-mm petri dishes in darkness for 24 h at 37 °C prior to experiments to reduce variability in larval nutritional state and physiological condition, thereby standardizing host status across experimental groups and facilitating reliable comparison of infection outcomes.

A preliminary experiment was conducted to determine the lethal dose of the CREC host strain used in the study. Larvae were injected with 10 μL of serial dilutions (1 × 10^2^–1 × 10^8^ CFU/ml) of the bacterial suspension to establish the optimal inoculum concentration for infection. Post-injection, larvae were incubated at 37 °C, and survival was monitored daily over a period of 120 h. The experiment was conducted in triplicate, with each replicate consisting of 10 larvae, following previously established protocols [30].

#### Experimental groups and phage therapy

Larvae were randomly assigned to four experimental groups (*n* = 10 per group), and all experiments were performed in triplicate. In the infection control group (IC), larvae were injected with a lethal dose of *E. coli* (1 × 10⁸ CFU/mL, 10 µL per larva). One hour after bacterial injection, 10 µL of sterile phosphate-buffered saline (PBS) was injected into a different (contralateral) last proleg of the same larva. In the phage therapy group (PT), larvae were first injected with the same lethal bacterial dose (*E. coli*, 1 × 10⁸ CFU/mL, 10 µL per larva). Subsequently, phage suspension was administered at a multiplicity of infection (MOI) of 10 (1 × 10⁹ PFU/mL, 10 µL per larva) into a different (contralateral) last proleg of the same larva 1 h after bacterial injection. The toxicity control group (Phage-Only Control-TC) received only the phage suspension (1 × 10⁹ PFU/mL, 10 µL per larva) in the absence of bacterial infection. One hour later, 10 µL of sterile PBS was injected into a different last proleg to control for the injection procedure. The negative control group (NC) was injected with sterile PBS (10 µL per larva), followed by a second injection of sterile PBS (10 µL) into a different last proleg 1 h later, to evaluate baseline survival and potential effects of repeated injection. All injections were performed using an insulin syringe via the last proleg, as previously described [31].

Larvae were placed into petri dishes and were incubated at 37 °C. *G. mellonella* larvae in the control, treatment, toxicity and infection groups were examined in detail at 24, 48, 72, 96 and 120 h for melanization, motility, cocoon formation and survival. *G. mellonella* health index scoring was used to compare the experimental groups [32]. All experimental groups were repeated in triplicate.

#### Microbiological analysis

To quantify the bacterial load of larva in IC and PT groups, four larvae from each group were sampled at each time point 0, 2-, 8-, 16-, and 24-h post-infection. The left prolegs of each larva were disinfected with 70% ethanol and gently pierced with a sterile needle to collect hemolymph. To prevent melanization, larvae were kept on sterile ice during sampling. On average, approximately 20 µL of hemolymph was obtained per larva. Hemolymph from each larva was processed individually, subjected to serial dilution in sterile physiological saline, and plated on EMB agar and blood agar. Plates were incubated at 37 °C for 24 h, after which colony-forming units (CFU) were counted to determine bacterial load [33, 34].

#### Histopathological analysis

Collected larvae were prepared for histological analysis by cryo-sectioning as previously described with slight modifications. The specimens were rapidly frozen using liquid nitrogen, and 7 μm-thick sections were obtained with a cryomicrotome (Cryostat/1850UV, LEICA) [35, 36]. The sections were then fixed in 100% acetone at room temperature. Hematoxylin and Eosin (H&E) staining was performed using a protocol optimized for frozen tissues. The sections were stained with Mayer’s hematoxylin and 1% eosin. The stained sections were dehydrated through a graded ethanol series, cleared in xylene, and mounted with Entellan [37, 38]. Tissue morphology was then examined under an optical microscope (Light microscope/DM3000, LEICA).

#### Statistical analysis

The survival curves were analyzed by the Kaplan–Meier method. For bacterial load, IC vs PT was compared separately at each time point using a two-sided Mann–Whitney U test. To control family-wise error across the five time-point comparisons, Holm’s adjustment was applied (α = 0.05). For interpretability, the group medians, the median log10 difference (IC–PT), and the median fold-reduction (IC/PT) calculated on the raw scale with a + 1 offset were reported. Data are visualized as box-and-whisker plots with individual observations overlaid. The *p* < 0.05 considered as significant.

## Results

### Phage biological characteristics

**Fig. 1:**
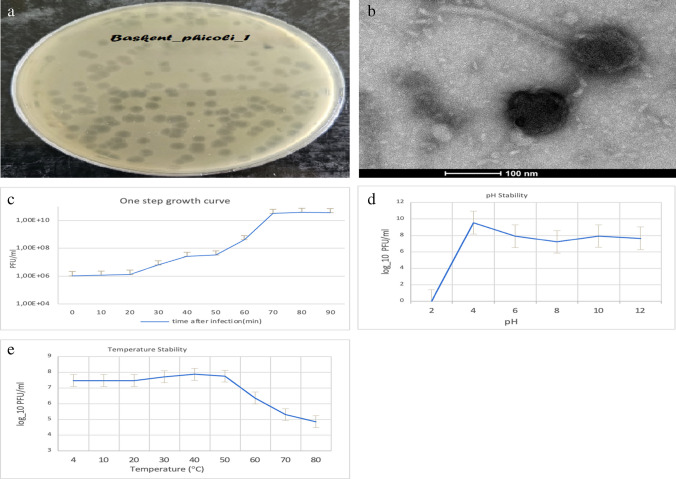
Biological properties of Baskent_phicoli_1 phage. **a.** Plaque morphology. **b.** The morphology of Baskent_Phicoli_1 as revealed by transmission electron microscopy. **c.** One-step growth curve. **d.** Stability of phage treated with different pH. **e.** Stability of phage at different temperatures. All experiment (for c, d, e) were performed in triplicate. Standard deviations were calculated, and error bars were given accordingly. Where error bars are not visible, it is because the values were too small

In the present study, a phage that exhibited complementary lytic activity against CREC-5 strain was isolated. This phage was designated as *Escherichia* phage Baskent_Phicoli_1. It was isolated from the wastewater. The Phicoli_1 phage formed small, clear, and round plaques approximately 2 mm in diameter (Fig. 1a).

The resulting micrographs from TEM revealed Baskent_phicoli_1 has icosahedral capsids approximately 70 nm in diameter and long, non-contractile tail approximately 210 nm in length consistent with siphovirus-like morphology as shown in Fig. 1b. These findings align with genome-based predictions, including the presence of a tape measure protein and other tail assembly genes typical of sipho-like phages. The optimal MOI for its host was 0.001 PFU/CFU. The burst size of phicoli_1 phage was calculated as 116, and the latent period was 20 min. One step growth curve was given in Fig. 1c. Evaluation of the stability of phages at different pH ranges showed that Phicoli_1 phage was stable when incubated for 60 min at pH 4–12; but there was no phage plaque at pH 2 (Fig. 1d). Thermal stabilities of phages were evaluated at temperatures from 4–80 °C, Phicoli_1 phage remained stable from 2 to 50 °C. with significant titer reduction observed at higher temperatures (> 60 °C) and completely lost its activity after 60 min of incubation at 80 °C (Fig. 1e). In the host range analysis, it was observed that the phage exhibited lytic activity only on its unique host strain, CREC-5 (ST1432), which was previously isolated from blood culture of a patients from Baskent University Adana Hospital. The sequence type (ST) of the isolate was determined as ST1432, and it was resistant to carbapenems (imipenem, meropenem and ertapenem), cephalosporins, gentamycin, ciprofloxacin while susceptible to amikacin and colistin. No activity was detected against the other clinical *E. coli* isolates tested. This finding indicates that the phage has a narrow host range.

### Phage genomic characteristic

In accordance with the International Classifcation Committee of Viruses, the phage belonged to the order *Caudovirales*, family *Autographiviridae*, subfamily *Studiervirinae*, genus *Kayfunavirus*. The genome of Baskent_phicoli_1 has been deposited in GenBank under Accession ID: PP766721. The genome of phage was double-stranded, circular, genome size of 39500 bp, and has 61 predicted coding sequences (CDS). The GC content of phage was 50.06%. No resistance genes, tRNAs, virulence, and lysogeny-related genes were detected in its genome.

**Fig. 2:**
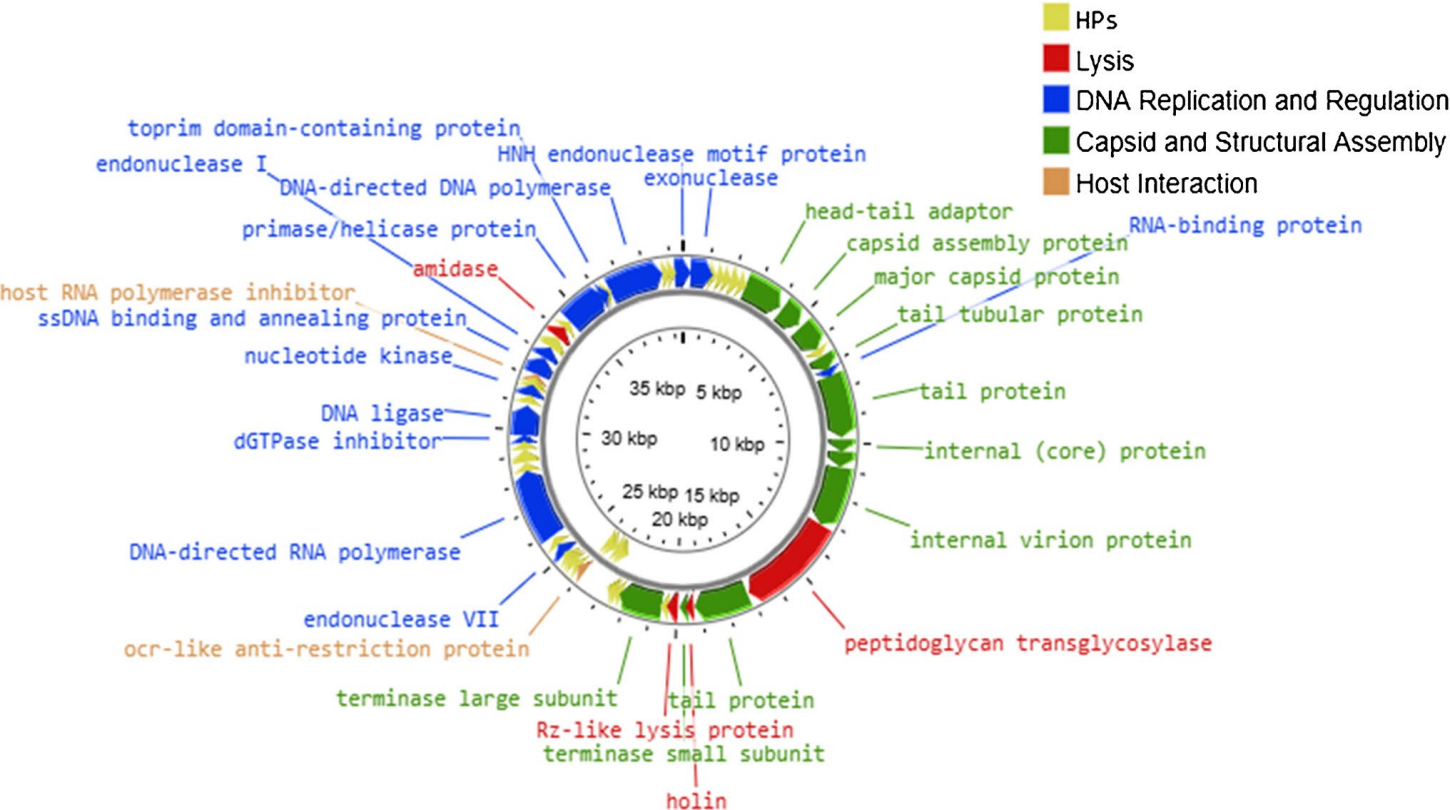
Genome map of Phicoli_1 phage. The genome of phage was depicted in circular. The arrows represent 61 CDS predicted the corresponding protein. HP: Hypothetical Protein

The genome map of the phicoli_1 phage reveals that it possesses well-organized genetic characteristics (Fig. 2). The phage genome was functionally divided into regions responsible for key biological processes, including DNA replication and regulation (blue), capsid and structural assembly (green), lysis (red), and host interaction (orange) while the remaining (yellow) indicates hypothetical proteins (HPs) (49.1%). The majority of genes associated with DNA replication and regulation, including DNA-directed DNA polymerase, primase/helicase, and exonuclease, are located between 24972 bp and 38686 bp, and these proteins are necessary for efficient phage propagation. The capsid and structural assembly genes, which include the major capsid protein (5244–6293 bp), tail tubular protein (6614–7180 bp), and internal virion proteins (10307–10894 bp), indicate a well-organized, complex structure that is required for host infection and genome packaging. The lysis-related genes, holin, a Rz-like lysis protein; peptidoglycan transglycosylase; and amidase, highlights the phage’s ability to lyse the bacterial cell wall for progeny release. The existence of various host interaction genes; host RNA polymerase inhibitors (31922–32080 bp), and anti-restriction protein (23942–24277 bp) implies the availability of strategies that facilitate the evasion of bacterial defense mechanisms.

**Fig. 3:**
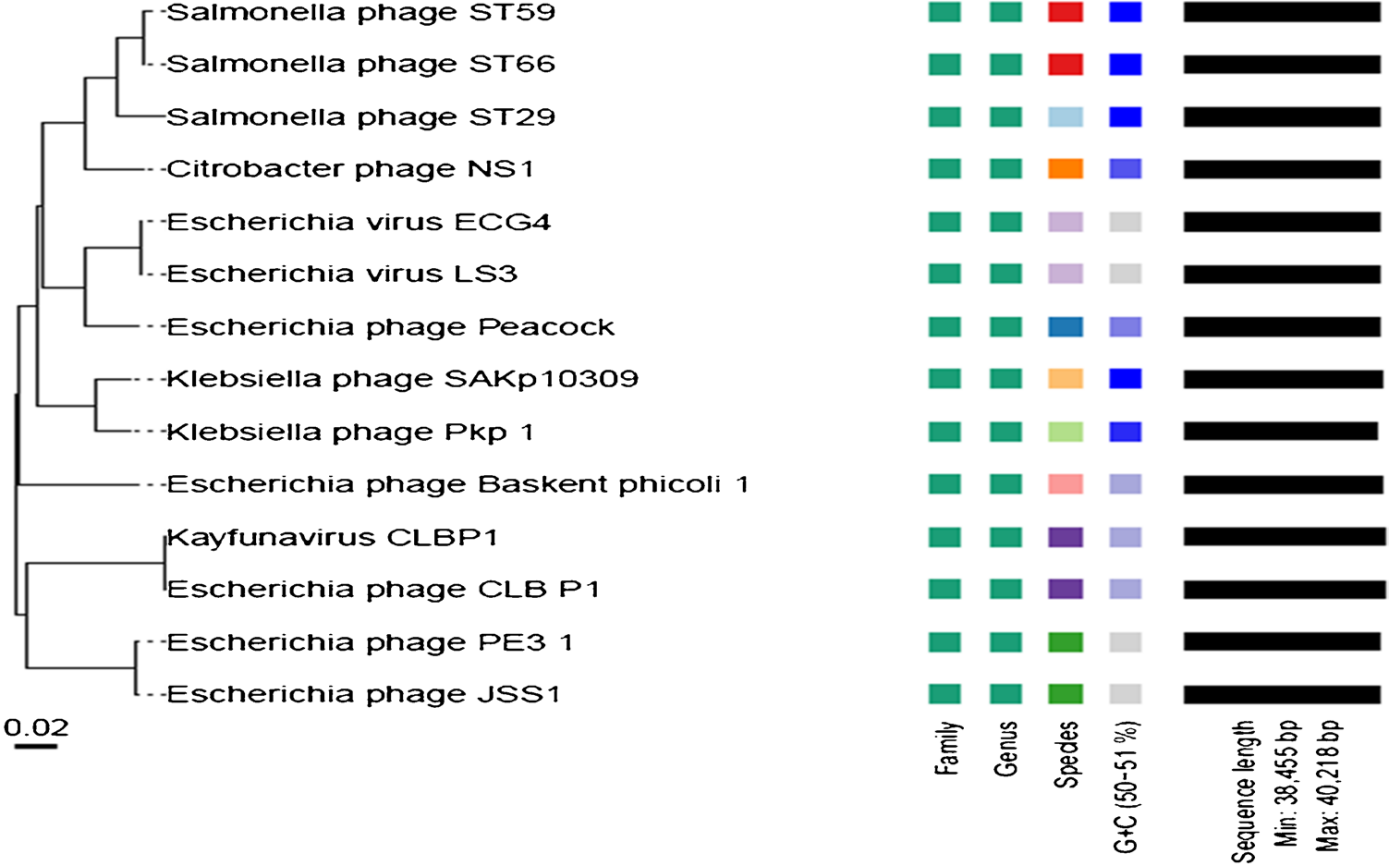
Phylogenetic trees of the Phicoli_1 phage based on whole genome sequences of. The tree was inferred using the formula D0 with a bootstrap support value from 100 replications. The branch lengths of the resulting VICTOR trees were scaled in terms of the respective distance formula used

The Genome-BLAST Distance Phylogenetic trees to determine the taxonomic relationship of the newly isolated phage was presented in Fig. 3. The generated tree revealed that our phage clustered most closely with *Escherichia* phages, including CLB P1, PES1, and JSS1, indicating a close evolutionary relationship within this group. More distant clustering was observed with *Salmonella* phages (ST59, ST66, ST29), *Citrobacter* phage NS1, and *Klebsiella* phages (SAKP10309 and Pkp1) formed a separate lineage. The topology was consistent with placement in the *Kayfunavirus* lineage, with Baskent_phicoli_1 branching as a distinct lineage relative to the closest *Escherichia* phages.

**Fig. 4:**
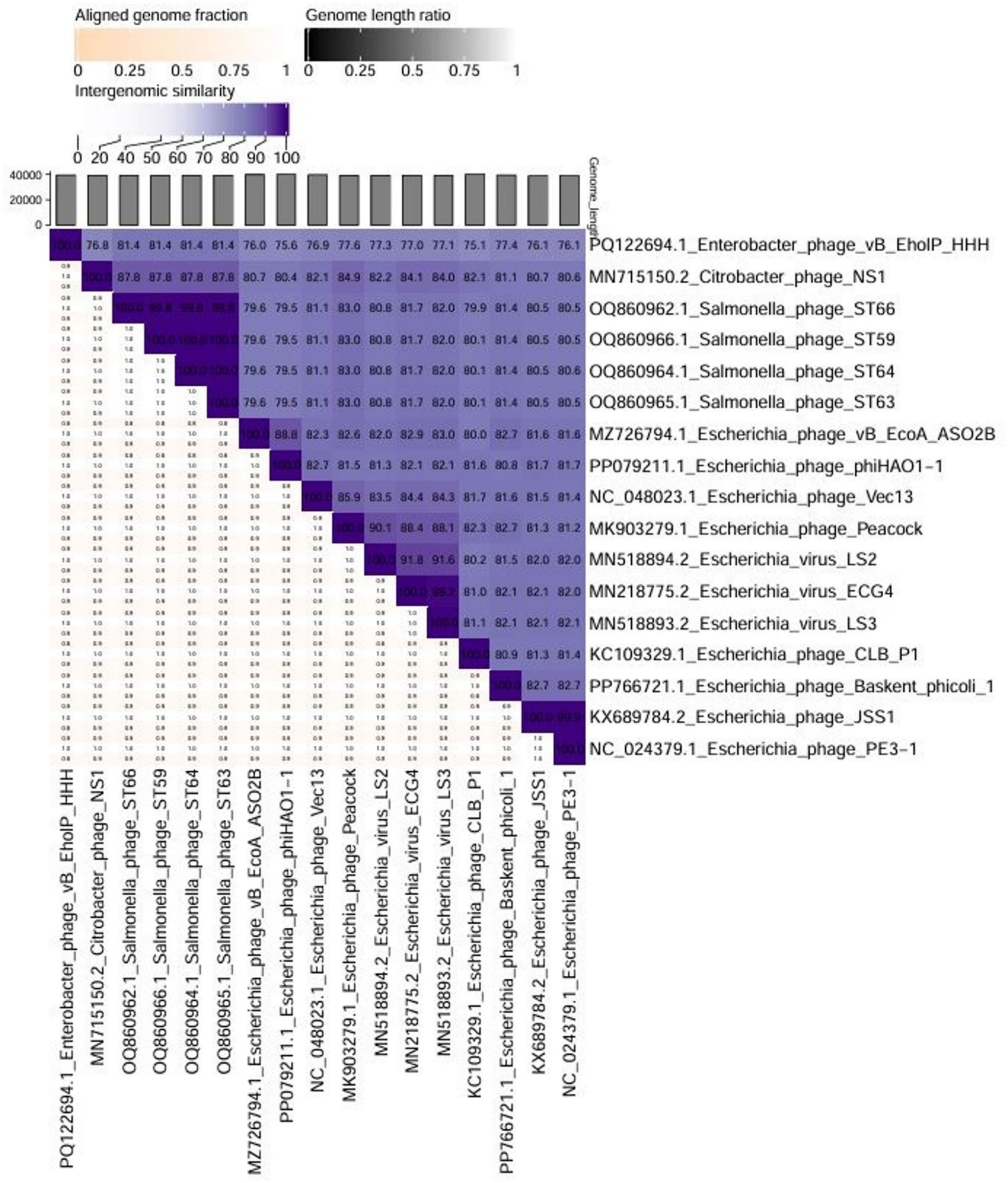
VIRIDIC analysis of Phicoli_1 phage with the most similar genome sequences based on BLASTn. The right part indicates the intergenomic similarity between pairs of sixteen phages, including phicoli_1 phage. The darker colors represent the greater percent of intergenomic similarity. The bars at the top represent the aligned genome fraction and genome length ratio for each pair of phages. A higher aligned genome fraction and genome length ratio indicate a larger portion of the genomes are aligned and the genome lengths are similar. The left part represents these related values, all ranked from 0 to 1

The VIRIDIC presents pairwise intergenomic similarities among a set of bacteriophage genomes, with values displayed as percentages (Fig. 4). In this dataset, several *Escherichia* and *Salmonella* phages show high levels of similarity, suggesting close evolutionary relationships. Conversely, lower similarity values between *Enterobacter, Citrobacter*, and *Salmonella* phages compared to the *Escherichia* phages indicate they belong to more distantly related taxa. The phage Baskent_phicoli_1 shows intergenomic similarity values below 82.7% with its closest relatives including *Escherichia* phages CLB P1, Peacock, and JSS1 and with all other phages in the dataset. This supports that Baskent_phicoli_1 belongs to the same genus (*Kayfunavirus*) as these *Escherichia* phages, but it represents a different novel species.

### In vivo efficacy

The therapeutic effect of Baskent_phicoli_1 phage against CREC infection in *G. mellonella* larval model was evaluated. The lethal dose was determined as 1 × 10⁸ CFU/ml. *G. mellonella* larvae in the control, phage treatment, toxicity and infection groups were examined at 24, 48, 72, 96 and 120 h in terms of melanization, motility, cocoon formation and survival.

**Fig. 5:**
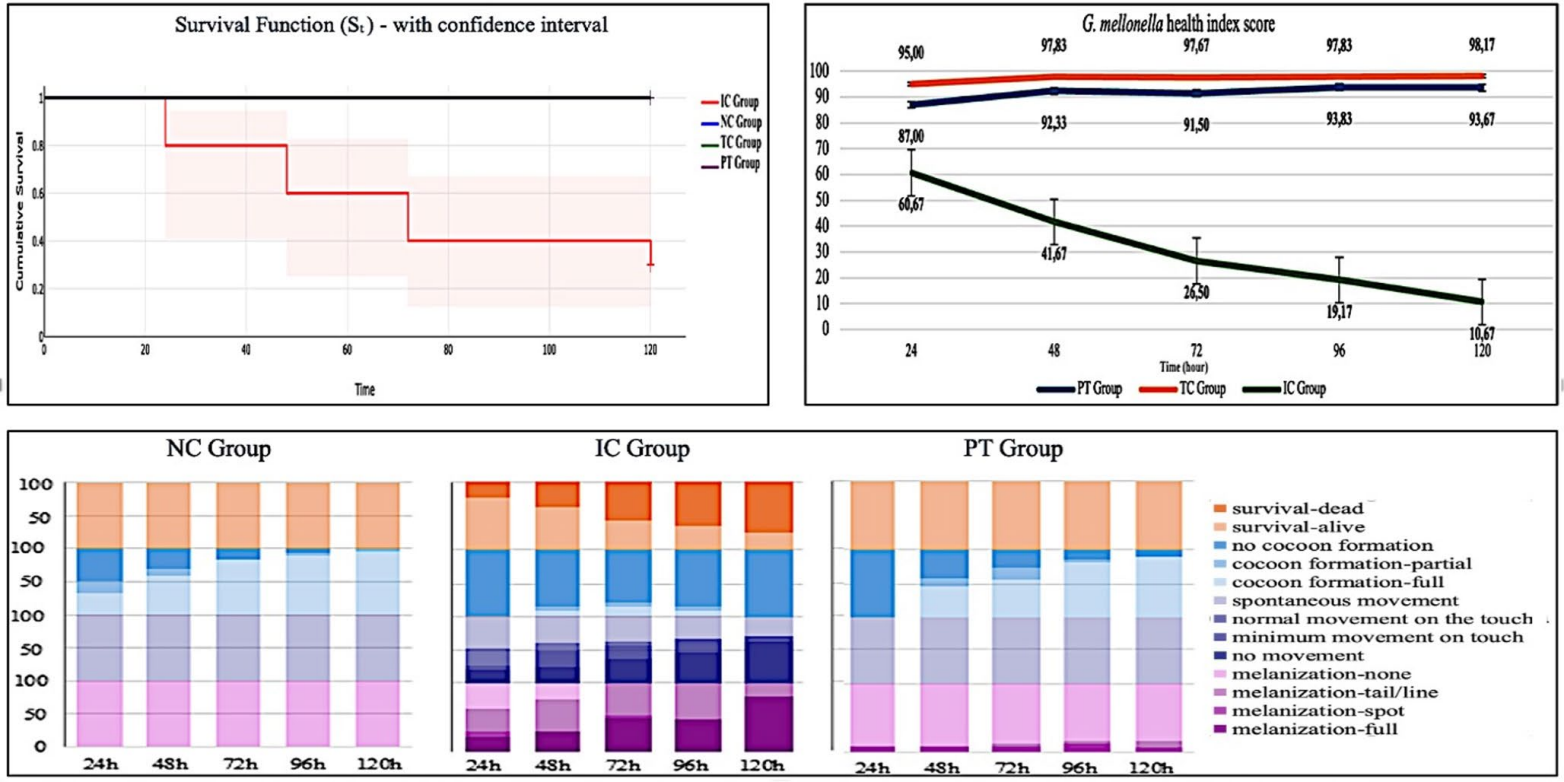
In vivo evaluation of phage therapy efficacy in the *Galleria mellonella* infection model. **a.** Kaplan Meier Survival analysis. Survival Function (St)- with confidence interval. The number of surviving larvae at 24, 48, 72, 96 and 120 h is plotted by Kaplan–Meier curve. Red: IC group, Green: TC, Blue: NC group, Purple: PT group. **b.** *Galleria mellonella* Health Index Scoring over time for comparing IC with PT and TC groups. **c.** Percentages of phenotypic observation of *G. mellonella* larvae post-infection with carbapenem-resistant *E. coli* ST1432, with and without phage therapy comparing to NC. Stacked bar graphs represent the distribution of scored phenotypes across timepoints (24 h, 48 h, 72 h, 96 h, 120 h) in three groups: Negative Control (NC), Infection Control (IC) Phage Therapy (PT) group. Each larva was scored for: Survival (Alive, Dead), Cocoon Formation (Full, Partial, None), Movement (Normal, Minimal, No movement), Melanization (None, Tail/line, Spots, Full body). Colors are grouped by category and standardized across both charts. Data represent the mean counts of scored larvae (*n* = 10 × 3 for each group, pooled from three independent experiments)

The IC group showed a decline in general health indicators (particularly in survival rate, and motility) over time compared to the PT and NC groups. The administration of phicoli_1 phage significantly improves larvae survival rate with 100% compared to the untreated IC group with 26.7% (*p* < 0.0001), indicating a strong therapeutic effect. The PT group, which had similar results to NC group, showed a strong response both physiologically (melanization) and behaviorally (movement, cocoon formation) compared to the IC. Moreover, phage administration was found to be non-toxic that phage therapy does not cause additional toxicity, as there was no statistical difference in survival between the PT and TC groups (*p* = 1.0), nor was there a significant difference with the TC and NC group. The survival of the untreated IC group was found to be significantly lower, thereby confirming the detrimental effect of CREC infection in *G. mellonella* and demonstrating the efficacy of phage therapy. As a result of Kaplan–Meier survival analysis using the log-rank (Mantel-Cox) test, it was determined that the survival rate in the phage treatment group was significantly higher than in the control group (*p* < 0.01) (Fig. 5a). After 72 h, the PT group showed a survival rate of 100%, whereas 73.3% of the untreated group died (*p* < 0.01).

PT and IC groups were compared in terms of larval activity, melanization, cocoon formation, movement and vital functions, and were presented in Fig. 5b. The treatment group scores followed a similar pattern to the toxicity control group demonstrating the treatment administered was safe. Scores in the toxicity control group were measured in the range of 95–98 at all time points. This indicates that the treatment did not cause any toxic effects on the larvae. Furthermore, the significant decrease in scores in the untreated infection group (IC) indicated that the infection model had been successfully established. In the phage treatment (PT) group, scores averaged 87 at 24 h and remained at 93.7 at 120 h. The high and stable scores indicate that the treatment protected the larvae from the effects of infection. In the infection control group, scores were 60.7 at 24 h and decreased to 10.7 at 120 h. The rapid decrease in scores revealed that the health status of the larvae deteriorated during the infection process and mortality increased.

In the untreated IC group, the most significant alterations in larval vital functions occurred at 48 and 72 h. The rate of melanization within the IC group increased progressively, with complete melanization (Fig. 5c) rising from 23.3% to 93.0% by 120 h. Mobility also declined over time in IC group, with 20.0% immobility at 24 h, increasing to 63.6% by 120 h. A significant reduction in responsiveness and increased immobility was observed at 48 and 72 h. Cocoon formation was markedly suppressed, with no cocoons present by 120 h in IC group. Survival decreased steadily from 80% at 24 h to 26.6% at 120 h, indicating a cumulative mortality of 73.3% in IC. The mortality rate was 0% for both NC and PT groups.

**Fig. 6:**
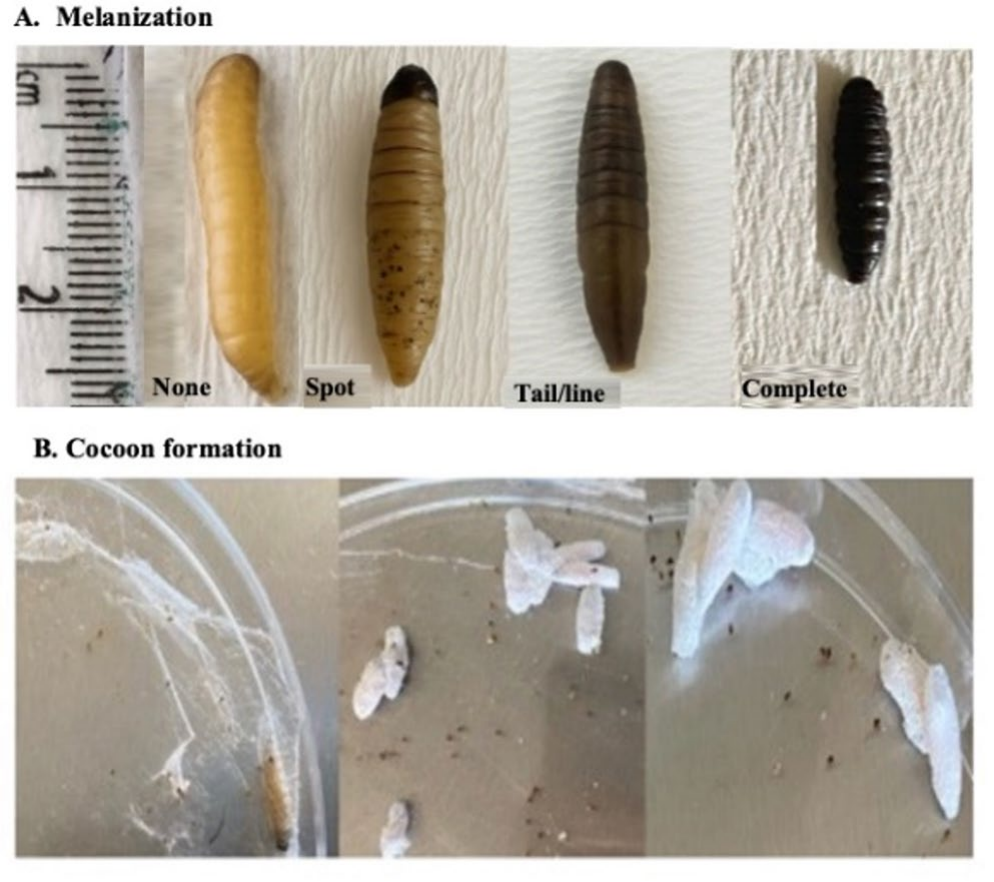
Morphology of *G. mellonella* larvae. **a.** Melanization. Changes in the appearance of *G. mellonella* larvae after infection indicated as complete, tail/line, spots compared to healthy final instar stage larvae in cream color without spots, while dead *G. mellonella* larvae were black. **b.** Cocoon Formation. Healthy larvae forming cocoons after treatment

On the other hand, in PT group, complete melanization was not observed, and 83.3% of larvae remained in the “none melanization” (Fig. 5c) category throughout the experiment, indicating effective protection against infection. All larvae maintained full mobility with 0% immobility across 5 days. Cocoon formation steadily increased, reaching 90.0% at 120 h, suggesting normal developmental progression. The survival rate remained at 100% at all-time points, with no mortality observed, clearly demonstrating the life-protective effect of the treatment. As seen in Fig. 5b and 5c, phage-treated larvae exhibited improved survival and cocoon formation with reduced melanization, indicating therapeutic efficacy. Figure 6a and 6b indicates the morphological changes in the appearance of *G. mellonella* larvae as complete, tail/line, spots compared to healthy final instar stage larvae, and healthy larvae forming cocoons after phage treatment.

### Microbiological analysis

**Fig. 7:**
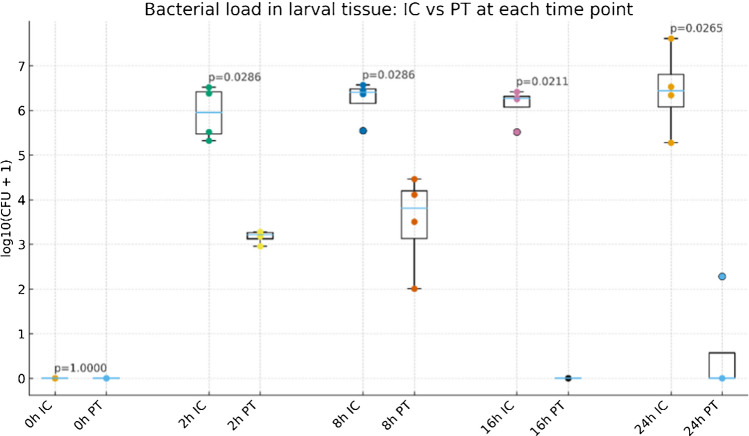
Bacterial load in infected larvae with and without phage therapy. Box-and-whisker plots of log10(CFU + 1) measured at 0, 2, 8, 16, and 24 h in untreated infection (IC) and Phage Therapy (PT) groups (*n* = 4 larvae per group per time). Boxes show the median and interquartile range; whiskers span the range; overlaid dots are individual larvae. Numbers above each IC–PT pair are two-sided Mann–Whitney U *p*-values computed on log10 (CFU + 1)

Bacterial burden (CFU per larva) was measured at 0, 2, 8, 16, and 24 h in Infection Control (IC) and Phage Therapy (PT) groups (*n* = 4 per group/time). Phage therapy rapidly and continuously reduced bacterial load compared with untreated infection group. With *n* = 4/group/time, median CFU fell from ~ 1.4 × 10^6^ in IC to ~ 1.7 × 10^3^ in PT at 2 h (≈8 × 10^2^-fold; ~ 2.9-log reduction), and from ~ 2.6 × 10^6^ to ~ 8.1 × 10^3^ at 8 h (≈3 × 10^2^-fold; ~ 2.5-log). By 16–24 h, PT was at/near the detection limit (0–190 CFU) while IC remained high (medians ~ 1.9–2.8 × 10^6^). Mann–Whitney tests on log10(CFU + 1) showed bacterial load was significantly higher than phage treated group at 2–24 h (*p* = 0.021–0.0286) as showed presented in Fig. 7, indicating a clear therapeutic impact of phage treatment on bacterial burden. The detailed statistical data was given in supplementary Table 1.

#### Histological examination

**Fig. 8:**
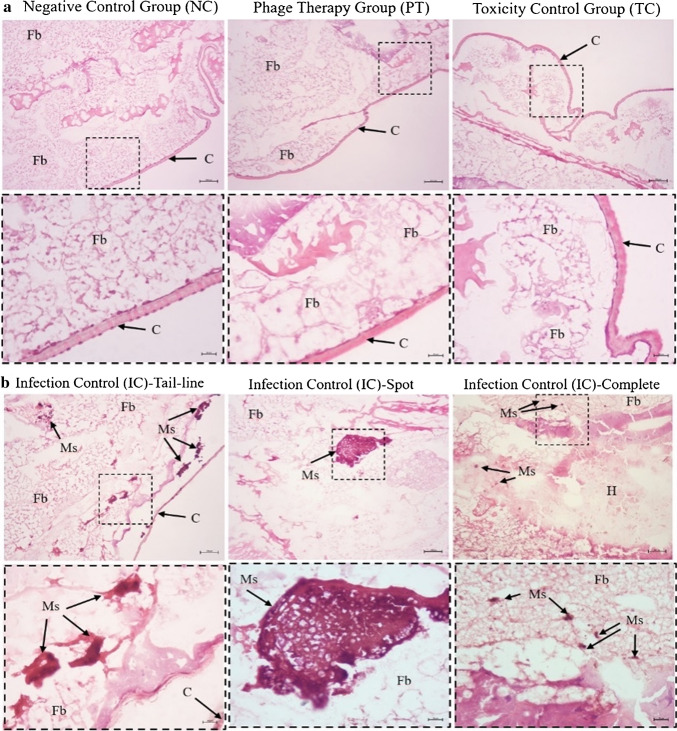
Histological features of *G. mellonella* larvae in different groups. **a.** The groups were negative control (phosphate-buffered saline (PBS) Control -NC), phage therapy (PT) and toxicity control (Phage-Only Control-TC). **b.** Histological evidence of melanization in *G. mellonella* larvae following infection with a lethal dose of *E. coli* (1 × 10⁸ CFU/ml) were given. Various forms of melanization were observed, indicating an immune response to bacterial infection. Key anatomical structures include the Cuticle (C), Fat body (Fb), Hemolymph (H), and Melanized structures (Ms). The tissue sections from infected larvae were stained with hematoxylin and eosin (H&E) and examined under a light microscope at 10 × magnification (upper row) and 40 × magnification (down row)

To assess the effects of phage therapy on larval tissues, histological analysis was performed on *G. mellonella* larvae from each experimental group. As shown in 8a, no melanization was observed in the NC group. Similarly, melanization was absent in tissue sections of both the PT group and the TC group, indicating no visible immune activation or tissue damage in response to phage administration (Fig. 8a). Additionally, the larval group that received post infection PT had significantly less tissue damage in the fat body and the muscle layer than the group IC. Furthermore, the TC group did not exhibit any tissue damage not also seen in the NC group (Fig. 8a). Extensive melanized nodules were observed in various regions of larval tissues from the IC group, which received a lethal dose of *E. coli* (1 × 10⁸ CFU/ml). Three distinct patterns of melanization were identified. In the tail-line pattern, melanization appeared in fragmented segments, limited to specific areas of the tissue. In the spot pattern, melanization was localized to a single region, forming a concentrated cluster. In the complete melanization type, widespread melanization was observed throughout most of the tissue in a scattered distribution (Fig. 8a-b).

## Discussion

*Escherichia coli* ST1432 is a multidrug-resistant strain with notable pathogenic potential, identified in both clinical infections and diverse environmental niches, emphasizing its role as a critical One Health concern and the need for coordinated eradication strategies targeting its persistence and transmission across human, animal, and environmental interfaces [39]. Notably, ST1432 belongs to clonal complex CC155, a lineage that has been widely associated with multidrug resistance and documented circulation across human, animal, and environmental reservoirs. The placement of ST1432 within CC155 underscores its epidemiological relevance, as this clonal complex is considered a key contributor to the persistence and cross-sector transmission of resistant *E. coli* within a One Health framework [40, 41]. The phage’s narrow host range, while limited in scope, could be beneficial by ensuring selective action against *E. coli* ST1432 and minimizing disruption of the normal microbiota. In addition, the limited number of clinical isolates included in the host range analysis represents a limitation of the present study. Phage therapy is increasingly implemented as a personalized, case-based approach, where narrow host range is an expected and often desirable feature. Such precision phages can be directly applied in isolate-specific treatments and may also serve as valuable components of rationally designed phage cocktails targeting multidrug-resistant pathogens. Studying phage therapy against such globally emerging sequence type is therefore crucial, offering a promising alternative to conventional antibiotics for controlling its spread and mitigating the risk it poses to public health and environmental safety.

Lytic bacteriophages, including Baskent_phicoli_1, offer valuable insights into phage-host interactions and present promising alternatives in the fight against antimicrobial resistance. The comprehensive genomic analysis of phicoli_1 reveals a highly specialized and evolutionarily adapted phage with no antimicrobial resistance-, virulence, and lysogeny-related genes. The current study highlights the genomic organization, functional capabilities, and therapeutic efficacy of this newly isolated and identified phage, positioning it as a promising candidate for future practical applications on demand.

The genomic organization of phicoli_1 phage indicates that it is a well-adapted lytic phage, possessing a specialized genetic background for infection, replication, and host cell lysis. The lysis-related genes of the phicoli_1 phage were found to contain a complete set of holin, Rz-like lysis protein, peptidoglycan transglycosylase, and amidase. This combination of genes is not commonly found in all phages. Lysis genes are critical for weakening the structural integrity of the bacterial cell wall and contributing to cell lysis [42]. Amidase mostly functions with other lytic proteins, including holins and lysozymes. Holins are responsible for creating pores in the membrane, thereby enabling amidase to access the peptidoglycan [42]. Lysozymes, also known as transglycosylases, are involved in the breakdown of glycosidic bonds within the peptidoglycan structure, thereby complementing the activity of amidase [43].

The Baskent_phicoli_1 phage genome encodes two remarkable host-interaction proteins—an RNA polymerase inhibitor and an OCR-like anti-restriction protein—both of which play crucial roles in overcoming bacterial defenses and facilitating successful phage replication. The first interacts with the bacterial RNA polymerase and suppress bacterial gene expression and shutting down host metabolism and defense mechanisms. Also, this protein redirects the host's transcriptional machinery toward expressing phage-specific genes. The OCR-like anti-restriction protein protects the phage genome from degradation by host restriction enzymes. This protein enables the phage to evade bacterial restriction-modification systems, a key defense mechanism of bacteria [44]. Its inhibition of restriction enzymes could provide insights into host–pathogen evolutionary interaction. Besides its biological significance, understanding how OCR-like proteins function could contribute to the development of advanced genome editing tools by helping to bypass bacterial restriction barriers. A notable feature of the phicoli_1 phage is the presence of its own machinery for initiating and regulating DNA synthesis, Toprim Domain-Containing Protein (36,215–36,304 bp) which is highly conserved motif involved in DNA replication and repair, making the phage less dependent on host replication enzymes [45, 46]. A considerable portion of the phicoli_1 genome encodes hypothetical proteins, representing genes whose functions remain uncharacterized based on current databases, highlighting areas for further investigation.

The intergenomic comparison of phicoli_1 (PP766721.1) against other phages reveals a maximum similarity of less than 82.7%, which falls below the 95% species demarcation threshold set by the International Committee on Taxonomy of Viruses (ICTV). According to ICTV guidelines, phages sharing less than 95% intergenomic similarity are typically classified as distinct species, indicating Baskent_phicoli_1 as novel species of *Kayfunavirus*. This uniqueness highlights its potential taxonomic and functional novelty within the analyzed phage group.

The in vivo infection model demonstrated the strong therapeutic efficacy of the phicoli_1 phage by markedly improved survival and developmental outcomes in treated larvae. Compared to the IC group—where larvae exhibited progressive melanization, reduced mobility, impaired cocoon formation, and high mortality—those receiving phage therapy maintained normal development and achieved a 100% survival rate. These results strongly suggest that phage therapy with Baskent_phicoli_1 not only reduces the negative effects of the bacterial infection but also preserves the host's physiological function. Comparable studies in invertebrate models, have shown similar protective effects of phage therapy against diverse bacterial pathogens. While this study provides valuable insights into the genomic structure and therapeutic potential of the Baskent_phicoli_1 phage by using in *G. mellonella* model, further validation in mammalian models, and as well as investigation optimal dosing regimens and the phage’s pharmacokinetics should be performed in further studies.

Our data show that phage therapy produced a rapid and durable reduction of bacterial burden in *G. mellonella* hemolymph relative to infection controls. Beginning at 2 h post-challenge, PT larvae carried ~ 10^2^–10^3^-fold fewer CFU than IC, and this advantage persisted at 8 h. By 16–24 h, PT counts were at or near the detection limit (0–190 CFU in all larvae), whereas IC remained heavily colonized with medians in the 10^6^ CFU range and occasional very high outliers. Nonparametric testing confirmed significant IC > PT differences at each post-infection time point (*p*≈0.02–0.03), the effect sizes were large and biologically compelling. Rapid decline followed by sustained suppression indicates efficient in-vivo phage attachment and replication, with amplification outpacing bacterial growth. The lack of rebound through 24 h suggests minimal emergence of phage-resistant bacteria and/or effective control by the host’s innate immunity acting with the phage. The marked therapeutic efficacy we observed is further supported by the intrinsic lytic properties of the phage used in this study. Its relatively short latent period (~ 20 min) combined with a high burst size (~ 116 progeny per infected cell) provides a strong kinetic advantage during infection. Such rapid amplification within the bacterial population, enable the phage to expand faster than *E. coli* replication. Taken together with the sustained suppression of bacterial loads and the lack of rebound over 24 h, these replication traits highlight the phage’s strong potential as a lasting and effective antibacterial therapy.

Histology supports the microbiological findings and provides context on safety. In larvae receiving phage alone (TC) or after infection (PT), sections showed no melanization and preserved architecture of the fat body (Fb) and muscle (Ms) layers, resembling the negative controls (NC). This pattern suggests that, at the administered dose and route, the phage preparation is not overtly tissue-toxic and does not provoke detectable activation of the melanization pathway over the sampling window. In contrast, the untreated IC group showed melanized nodules with multifocal tissue disruption, indicating an active inflammatory response to the high bacterial load. In the IC group, the three melanization patterns—tail-line, spot, and complete—likely indicate increasing infection severity and spread, progressing from small, localized areas to widespread involvement. Because the PT group lacked these histologic changes and showed much lower CFU counts, phage therapy likely limited the bacterial growth and the release of pro-inflammatory products, thereby reducing tissue damage. This analysis is limited by the semi-quantitative nature of H&E. Histological findings provide descriptive evidence of tissue-level effects. The lack of quantitative histopathological scoring represents a limitation of the present study. Future studies should incorporate blinded scoring, quantitative measures (e.g., melanization area, hemocyte counts, phenoloxidase activity), and time-course sampling to link phage exposure, bacterial load, and host responses [32, 47, 48]. Overall, treated larvae show preserved tissue with no apparent phage-related toxicity, while damage is largely confined to untreated infection group.

Although the *Galleria mellonella* infection model is widely used for initial assessment of microbial virulence and preclinical efficacy due to its simplicity, rapidity, and cost-effectiveness, it has notable limitations compared with mammalian systems. Unlike mammalian hosts, *G. mellonella* lacks adaptive immunity, and its genome is not fully sequenced, with limited genetic tools available for mechanistic studies. In addition, variability in larval source, breeding conditions, maintenance, and handling can influence susceptibility to infection and immune responses, complicating reproducibility across laboratories. For example, differences in supplier, storage conditions, and post-infection incubation have been shown to yield divergent mortality outcomes with the same bacterial strains. These limitations highlight the need for caution in extrapolating results directly to clinical contexts and support the use of *G. mellonella* as a preclinical proof-of-concept model rather than a substitute for mammalian infection models [32].

## Conclusion

In conclusion, this study successfully isolated and characterized a novel bacteriophage, Baskent_phicoli_1 with promising characteristics for biotechnological applications, coupled with stability and rapid lytic activity against CREC. Comprehensive genome analysis revealed the absence of any antimicrobial resistance, virulence, and lysogeny related genes, further supporting its potential as a safe therapeutic agent. Notably, the phage harbors genes encoding crucial host defense modulators which enables the phage to overcome bacterial restriction-modification systems, ensuring successful genome propagation. The in vivo assessment using the *Galleria mellonella* infection model revealed the phage's remarkable therapeutic efficacy, evidenced by a 100% survival rate in the treatment group compared to the 73.3% mortality in the untreated control. Furthermore, histological analysis corroborated the protective effects of phage therapy, showing significantly reduced tissue damage in the treated larvae.

The combination of lytic activity supports the potential of phage Baskent_phicoli_1 as a strain-specific, preclinical proof-of-concept, rather than a stand-alone therapeutic, against CREC infections. This study provides preclinical evidence for a strain-specific lytic bacteriophage targeting carbapenem-resistant *Escherichia coli* ST1432, an epidemiologically relevant lineage of clinical concern. Future studies should validate these findings in mammalian infection models, evaluate pharmacokinetic and immunogenic properties, and assess the phage within rationally designed phage cocktails to clarify its translational potential beyond the preclinical setting.

## Supplementary Information

Below is the link to the electronic supplementary material.

Supplementary file1 (DOCX 28 KB)

## Data Availability

Sequence data that support the findings of this study has been deposited in GenBank of National Library of Medicine (https://www.ncbi.nlm.nih.gov/) with the primary accession number of PP766721 and PRJNA1330585.
