# Supplementary material for: A novel phage encoding host defense modulators demonstrates in-vivo therapeutic efficacy against carbapenem-resistant E. coli ST1432
Source: Eur J Clin Microbiol Infect Dis. 2026 Feb 23;45(6):1583–97. doi: 10.1007/s10096-026-05422-7 (PMC13319148; doi:10.1007/s10096-026-05422-7)
Supplement: Supplementary file 1 — Supplementary file1 (DOCX 28 KB) [file 10096_2026_5422_MOESM1_ESM.docx]

# Supplementary Table S1. Bacterial load in larval tissue following phage therapy

| **Time point** | **IC median log₁₀(CFU + 1)** | **PT median log₁₀(CFU + 1)** | **Median log₁₀ difference (IC–PT)** | **Median fold reduction (IC/PT)** | **p value** |
| --- | --- | --- | --- | --- | --- |
| 0 h | 0.000 | 0.000 | 0.000 | 1.0 | 1.0000 |
| 2 h | 5.474 | 4.188 | 1.287 | 1.93 × 10¹ | 0.0286 |
| 8 h | 5.740 | 4.250 | 1.490 | 3.09 × 10¹ | 0.0286 |
| 16 h | 6.270 | 0.000 | 6.270 | 1.86 × 10⁶ | 0.0211 |
| 24 h | 7.305 | 0.000 | 7.305 | 2.02 × 10⁷ | 0.0265 |

Bacterial loads are expressed as log₁₀(CFU + 1). IC (untreated infection) and PT (phage therapy) groups were compared independently at each time point using a two-sided Mann–Whitney U test (n = 4 larvae per group per time point). To control for multiple comparisons across five time points, Holm’s adjustment was applied (family-wise α = 0.05). P values are derived from comparisons of individual larval measurements; none remained statistically significant after Holm correction.
